# Enhanced mitochondrial function in B cells from elderly type-2 diabetes mellitus patients supports intrinsic inflammation

**DOI:** 10.3389/fragi.2024.1444527

**Published:** 2024-08-23

**Authors:** Daniela Frasca, Valquiria Bueno

**Affiliations:** ^1^ Department of Microbiology and Immunology, University of Miami Miller School of Medicine, Miami, FL, United States; ^2^ Sylvester Comprehensive Cancer Center, University of Miami Miller School of Medicine, Miami, FL, United States; ^3^ Department of Microbiology Immunology and Parasitology, UNIFESP, Federal University of São Paulo, São Paulo, Brazil

**Keywords:** aging, B cells, inflammation, metabolism, type-2 diabetes mellitus

## Abstract

In this paper, we measured B cell function in elderly healthy individuals (E_H_) and in elderly patients with Type-2 Diabetes Mellitus (T2DM, E_T2DM_), which are treatment-naive, as compared to healthy young (Y_H_) individuals. Results show a higher serum inflammatory status of elderly versus young individuals, and especially of E_T2DM_ versus E_H_. This status is associated with a reduced response to the seasonal influenza vaccine and with increased frequencies of the circulating pro-inflammatory B cell subset called Double Negative (DN) B cells. B cells from E_T2DM_ patients are not only more inflammatory but also hyper-metabolic as compared to those from E_H_ controls. The results herein are to our knowledge the first to show that T2DM superimposed on aging further increases systemic and B cell intrinsic inflammation, as well as dysfunctional humoral immunity. Our findings confirm and extend our previously published findings showing that inflammatory B cells are metabolically supported.

## Introduction

Aging is associated with a chronic low-grade systemic inflammatory status, known as inflammaging, which is known to be involved in the establishment and in the pathogenesis of several debilitating diseases typical of old age, including Type-2 Diabetes Mellitus (T2DM), a disease characterized by obesity, hypertension, dyslipidemia, accelerated atherosclerosis and increased mortality (Alberti and Zimmet, 1998). T2DM is generally not associated with autoimmunity (https://doi.org/10.2337/dc14-S081). Inflammaging supports T2DM through the acquisition of the senescence-associated secretory phenotype (SASP) by immune and non immune cells, as well as through oxidative stress and endoplasmic reticulum stress (Prattichizzo et al., 2016), two processes associated with advanced age (Muriach et al., 2014; Bonomini et al., 2015). Life expectancy of T2DM patients has been reported to be about 5–10 years shorter than that of age-matched healthy controls, and in general it has been shown that earlier a person is diagnosed with T2DM greater the reduction in life expectancy is. This is mainly due to the increased risk for progressive disability due to T2DM-associated chronic conditions, as well as to vascular complications, such as heart attack, stroke and aneurysms, as well as increased age (Huang et al., 2014).

T2DM patients have dysfunctional immune responses (Geerlings and Hoepelman, 1999; Ferlita et al., 2019; Frasca et al., 2021a), leading to increased susceptibility to infections (Casqueiro et al., 2012; Carrillo-Larco et al., 2022) and reduced responses to vaccination (Frasca et al., 2021a; Xiang et al., 2023) as compared to healthy age-matched controls. These defects are exacerbated in older T2DM patients, because of their increased inflammatory condition (Pozzilli et al., 1986; Smith and Poland, 2000; Muller et al., 2005; McElhaney et al., 2015). One of the major causes of dysfunctional immunity in T2DM patients is hyperglycemia (Berbudi et al., 2020), which derives from to both insufficient insulin action (insulin resistance) and impaired insulin production by the β cells of the pancreas, with consequent increased glucose levels in the blood. Hyperglycemia induces intrinsic inflammation in cells of the innate and adaptive immune system, which is known to be associated with decreased cell function.

Treatment of T2DM patients includes intensive lifestyle modifications with the goal of glycemic and cardiovascular risk factor management, weight management through medical nutrition therapy and diet, as well as pharmacological treatments such as metformin (https://www.uptodate.com/contents/initial-management-of-hyperglycemia-in-adults-with-type-2-diabetes-mellitus). Metformin is currently the only drug with hypoglycemic effects also able to counteract inflammatory processes associated with the development of chronic conditions of old age. Our previously published results have shown that metformin induces optimal B cell function and humoral immunity to the seasonal influenza vaccine in elderly individuals through decreased intrinsic B cells inflammation and metabolic pathways that B cells utilize to support the secretion of inflammatory mediators and pathogenic autoimmune antibodies (Frasca et al., 2021a).

In this paper, we evaluated B cell function in elderly healthy individuals (E_H_) and in elderly T2DM patients (E_T2DM_), which are treatment-naive, as compared to healthy young (Y_H_) individuals. Results herein show a higher serum inflammatory status of elderly versus young individuals, and especially of E_T2DM_ versus E_H_, showing for the first time that T2DM superimposed on aging further increases inflammaging and dysfunctional humoral immunity. This status is associated with a reduced response to the influenza vaccine and increased frequencies of circulating Double Negative (DN) B cells, which represent the most pro-inflammatory B cell subset. Moreover, B cells are more inflammatory and more metabolically supported in E_T2DM_ versus E_H_.

## Materials and methods

### Subjects

We recruited the following groups of overweight participants (body-mass index, BMI, 25–29.9 kg/m^2^): elderly patients with T2DM (E_T2DM_) (n = 10, age 72 ± 2, BMI = 26.18 ± 0.94) or without (n = 10, age 74 ± 1, BMI = 27.03 ± 0.41), as well as healthy young individuals (Y_H_, n = 10, age 35 ± 1, BMI = 27.28 ± 0.33) as controls. All T2DM patients are treatment-naïve. Both T2DM patients and healthy participants were recruited at the University of Miami Miller School of Medicine. Participants were screened for consumption of medications, drugs or alcohol, all known to alter immune responses as well as for diseases such as autoimmune diseases, malignancies, cardiovascular, renal or hepatic diseases, infectious disease, trauma or surgery, pregnancy. Participants signed an informed consent. The study was reviewed and approved by our Institutional Review Board (IRB, protocols #20070481 and #20160542), which reviews all human research conducted under the auspices of the University of Miami.

### Influenza vaccination

Participants were recruited during the 2013–2014 influenza vaccine season. The 2013–2014 vaccine contained A/California/7/2009 (H1N1), A (H3N2) virus antigenically like the cell-propagated prototype virus A/Victoria/361/2011 and B/Massachusetts/2/2012. Participants were influenza-free at the time of recruitment and without any symptom of respiratory tract infections. Blood samples were collected immediately before (T0) and 28 days (T28) after the influenza vaccine, to capture baseline and late antibody responses, respectively.

### Hemagglutination inhibition (HAI) assay

We measured influenza vaccine-specific serum titers at T28 by Hemagglutination Inhibition Assay (HAI), which is the best correlate for vaccine protection and represents the only measure for vaccine efficacy (Murasko et al., 2002; Skowronski et al., 2008; Frasca and Blomberg, 2020).

### PBMC collection

Blood was drawn in Vacutainer CPT tubes (BD 362761). PBMC were isolated and cryopreserved. On the day of the experiment, PBMC (1 × 10^6^/mL) were thawed, rested, checked for viability and cultured in complete medium (c-RPMI, RPMI 1640, supplemented with 10% FCS, 10 μg/mL Pen-Strep, 1 mM Sodium Pyruvate, and 2 × 10^−5^ M 2-ME and 2 mM L-glutamine). Samples with less than 75% viability, evaluated by trypan blue counting, were not used.

### B cell isolation and *in vitro* stimulation

B cells were isolated from thawed PBMC by positive selection using CD19 magnetic beads (Miltenyi 130-050-301), following manufacturer’s instructions. Cell preparations were typically >95% pure. For *in vitro* cytokine secretion, B cells were stimulated for 2 days in c-RPMI with CpG (invivoGen ODN 2006, 2 µg/10^6^ B cells/mL), then supernatants were collected and stored until use.

### Flow cytometry

After thawing, PBMC (2 × 10^6^/mL) were stained for 20 min at room temperature with Live/Dead kit and the following antibodies: anti-CD45 (Biolegend 368540), anti-CD19 (BD 555415), anti-CD27 (BD 555441) and anti-IgD (BD 555778) to measure naive (IgD+CD27−), IgM memory (IgD+CD27+), switched memory (IgD-CD27+), and DN (IgD-CD27−) B cells. For the evaluation of cytosolic ROS, PBMC were stained with the CellROX^®^ Deep Red Reagent (Thermo Fisher C10422), whereas for the evaluation of mitochondrial ROS we used the MitoSOX™ Red Reagent (mitochondrial superoxide indicators, Thermo Fisher M36009). For mitochondrial mass we used MitoTracker Green (Thermo Fisher M7514). Working concentrations were recommended by the manufacturers. Staining was done at room temperature for 30 min. Cells were then washed and then membrane stained with the antibodies above for additional 20 min at room temperature.

To detect plasmablasts (CD3^−^CD19^+^CD20^low^CD27^bright^CD38^bright^), we stained PBMC with anti-CD3 (BD 555339), anti-CD19 (BD 555415), anti-CD20 (BD 641396), anti-CD27 (BD 555441), anti-CD38 (BD 551400). Up to 10^5^ events in the B cell gate were acquired on an LSR-Fortessa (BD) and analyzed using FlowJo 10.5.3 software. In every experiment we included single color controls for compensation purposes as well as isotype antibodies to set up the gates.

### Sorting of DN B cells

DN B cells were sorted in a Sony SH800 cell sorter using anti-CD45, anti-CD19, anti-CD27 and anti-IgD antibodies. Cell preparations were typically >98% pure. Sorted DN B cells were stimulated with CpG for 48 h, then supernatants were collected and evaluated for cytokine secretion by CBA.

### *In vitro* secretion of cytokines and metabolites

Culture supernatants of sorted CpG-stimulated cells were collected after 48 h to measure the secretion of pro- and anti-inflammatory cytokines, and of the metabolite lactate, by flow cytometry using the BD™ Cytometric Bead Array (CBA) human TH1/TH2/TH17 kit (BD 560484), or by ELISA using the L-lactate assay kit (Cayman Chemicals 700510), respectively.

### mRNA extraction and quantitative (q)PCR

To evaluate mRNA expression of glucose transporter 1 (Glut1) and of Pyruvate DeHydrogenase X (PDHX), the mRNA was extracted from B cells, using µMACS magnetic beads (Miltenyi 130-122-752) following manufacturer’s instructions. The mRNA was eluted into 75 µL of pre-heated (65°C) elution buffer, and stored at −80°C until use. Reverse Transcriptase (RT) reactions were performed in a Mastercycler Eppendorf Thermocycler to obtain cDNA. Briefly, 10 µL of mRNA + 10 µL of RT-mix were used for cDNA synthesis. Conditions were: 40 min at 42°C and 5 min at 65°C.

Five µL of cDNA were used for qPCR. Reactions were conducted in MicroAmp 96-well plates and run in the ABI 7300 machine. Calculations were made with ABI software. For calculations, we determined the cycle number at which transcripts reached a significant threshold (Ct) for both target genes and for GAPDH (control). The difference in Ct values between GAPDH and each of the target genes was calculated as ΔCt. Then the relative amount of the target gene was expressed as 2^−ΔCt^ and indicated as qPCR values. Reagents and Taqman primers, all from ThermoFisher, were the following: GAPDH, Hs99999905_m1; Glut1 (SLC2A1), Hs00892681_m1; PDHX, Hs00185790_m1.

### ELISA

Serum levels of TNF-α, IL-6, C-reactive protein (CRP), Serum Amyloid A (SAA) were measured by the commercially available kits KHC3014, KHC0061, KHA0031, KHA0011, respectively, all from ThermoFisher Scientific. Lactate Dehydrogenase (LDH) was measured by the commercially available kit ab102526 (abcam).

### Statistical analyses

To examine differences between two groups, unpaired Student’s t tests (two-tailed) were used. To examine relationships between variables, bivariate Pearson’s correlation analyses were performed. Principal Component Analyses (PCA) were generated using Prism version 10.1.0 software, which was also used to construct all graphs.

## Results and discussion

### E_T2DM_ patients have higher levels of serum markers of inflammaging as compared to E_H_ individuals

**FIGURE 1:**
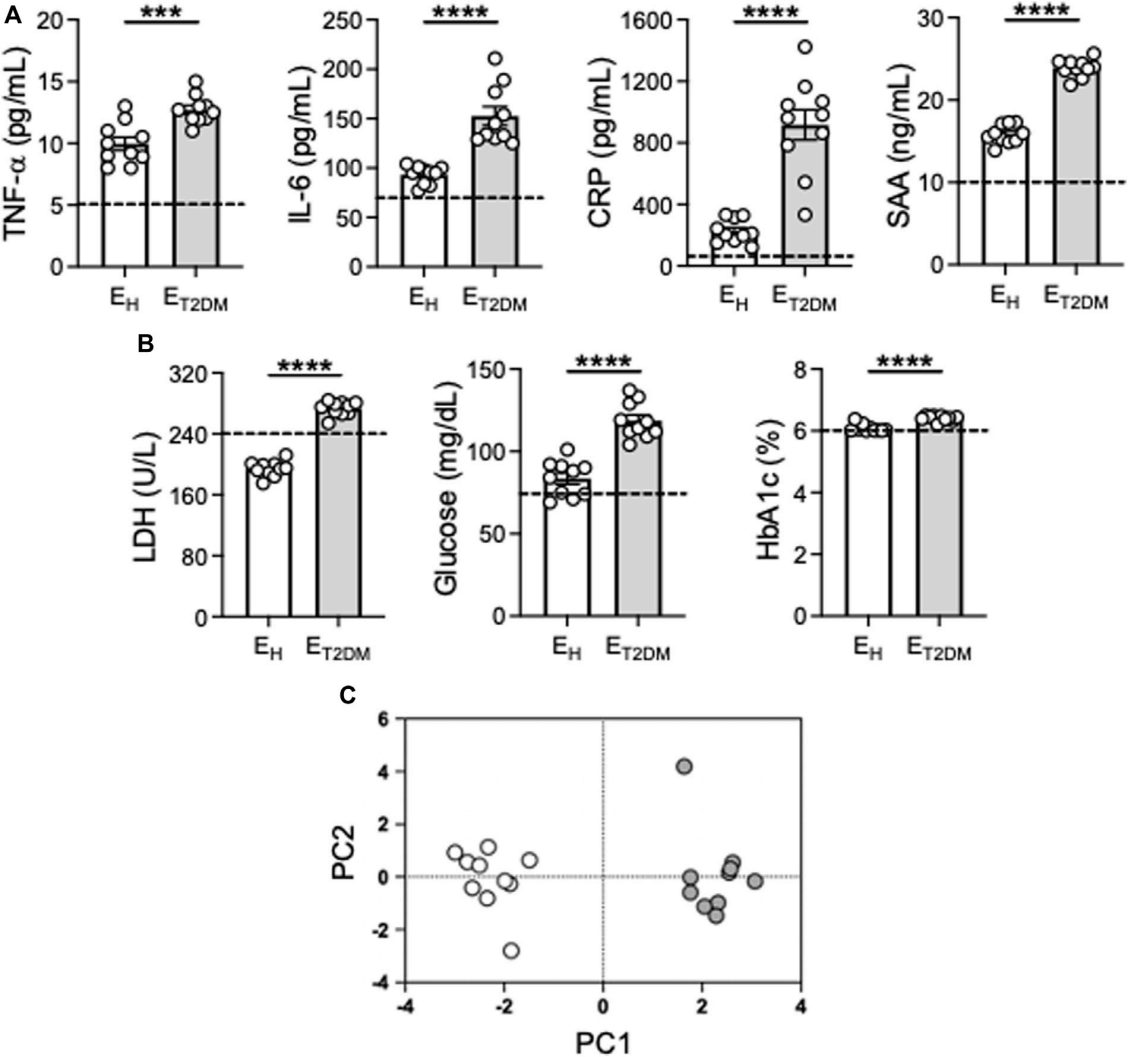
Increased levels of serum markers of inflammaging in E_T2DM_ patients as compared to E_H_ individuals. Serum samples were collected from E_H_, E_T2DM_ and young controls and evaluated for the presence of the indicated markers by ELISA **(A, B)**. Dotted lines indicate values in young individuals (TNF-α: 5.1 ± 0.7; IL-6: 70.5 ± 4.1; CRP: 85.1 ± 8.7; SAA: 10.6 ± 1.8; Glucose: 74.8 ± 3.2; Hb1ac: 5.9 ± 0.3), LDH: 239.4 ± 31.2. **(C)** PCA analyses with the axes showing the percentage of variation explained by PC1 and PC2. Each symbol corresponds to one individuals. Open circles, E_H_; filled circles, E_T2DM_. Mean comparisons between groups were performed by unpaired Student’s t test (two-tails). ***p < 0.001, ****p < 0.0001.

We evaluated the serum inflammatory profile of the participants. We measured serum levels of the pro-inflammatory markers TNF-α, IL-6, CRP, SAA, all associated with inflammaging, as well as of the metabolic markers glucose, glycated hemoglobin 1c (HbA1c) and LDH. These inflammatory/metabolic markers have been selected because they are among those that have been used to estimate the biological age of elderly individuals (≥65 years) (Verschoor et al., 2022), and also because they are elevated in patients with metabolic disorders, including those with T2DM (Hsieh et al., 2022). Results in Figure 1A show higher levels of all these markers in elderly versus young individuals (dotted line), with levels in E_T2DM_ patients being significantly higher than those in E_H_ controls. PCA analyses and distinct clustering of serum inflammatory/metabolic markers in the three groups of participants are shown in Figure 1C.

These results clearly demonstrate that T2DM superimposed on aging further exacerbates the inflammatory status of the individuals which is central to pathogenic processes in T2DM and metabolic syndrome, leading to the well known complications of the disease as those damaging blood vessels in the eyes, causing permanent vision loss, or those damaging blood vessels in the kidneys, nerves or in the heart (Galicia-Garcia et al., 2020).

### E_T2DM_ patients have lower antibody responses to the seasonal influenza vaccine as compared to E_H_ individuals

**FIGURE 2:**
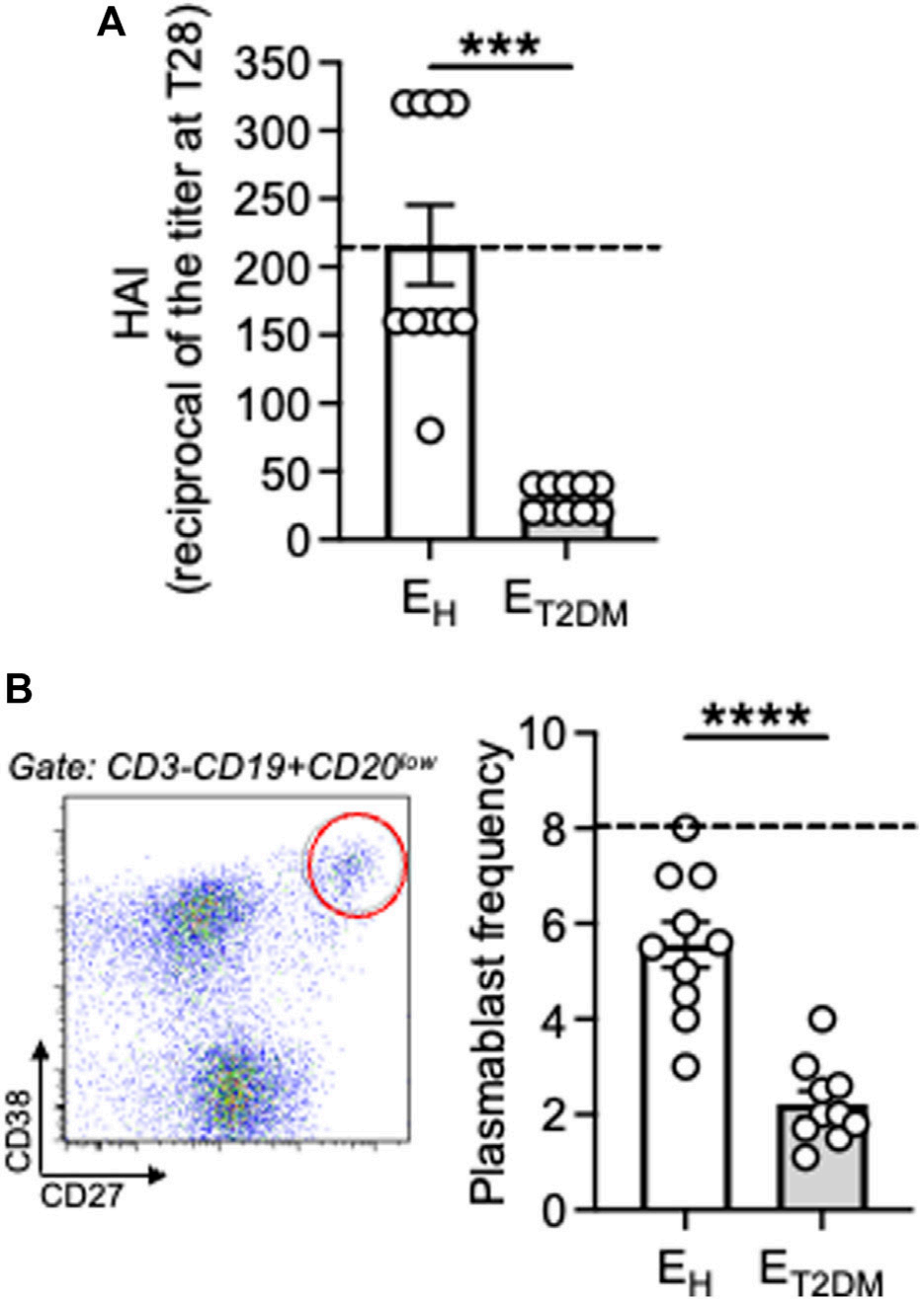
Decreased antibody responses to the seasonal influenza vaccine in E_T2DM_ patients as compared to E_H_ individuals. **(A)** Serum samples were collected from E_H_, E_T2DM_ and young controls. Vaccine-specific antibodies were evaluated by HAI. Results show the reciprocal of the titers at T28. Dotted lines indicate values in young individuals (216 ± 54). **(B)** PBMC at T7 after influenza vaccination were membrane stained with antiCD3/CD19/CD20/CD27/CD38 antibodies to detect plasmablasts. Left. A representative staining is shown for one E_H_ individual. Right. Frequencies of plasmablasts from all individuals (CD3^−^CD19^+^CD20^low^CD27^bright^CD38^bright^). Dotted lines indicate values in young individuals which were 8 ± 1. Mean comparisons between groups were performed by unpaired Student’s t test (two-tails). ***p < 0.001.

We then evaluated the antibody response to the influenza vaccine in the same three groups of participants. We measured vaccine-specific antibody titers in serum by HAI, which is the best correlate for vaccine protection and represents the only measure for vaccine efficacy (Murasko et al., 2002; Skowronski et al., 2008; Frasca and Blomberg, 2020). Results in Figure 2A show that vaccine-specific antibodies were decreased by both aging and T2DM. All E_H_ individuals, but only half of E_T2DM_ patients, showed increased titers at T28 as compared to baseline.

We also measured by flow cytometry the plasmablast response at T7 after influenza vaccination in the same subjects above. We (Frasca et al., 2016a) and others (Wrammert et al., 2008; Sasaki et al., 2011) have shown that this is the peak for the plasmablast response, which is completely gone at T14, and this response at T7 is vaccine-specific. Results in Figure 2B show that vaccine-specific plasmablasts were also decreased by both aging and T2DM. These results altogether confirm and extend previously published observations on age-effects of influenza vaccine-induced humoral immunity [reviewed in Frasca and Blomberg (2020), Frasca et al. (2020)] and on the effects of T2DM on these responses (Frasca et al., 2021a). Not only the antibody response to the influenza vaccine is decreased in elderly individuals with T2DM, also the antibody response to COVID vaccines has been shown to be impaired, as recently published in a systematic review (He et al., 2023).

### The B cell pool of E_T2DM_ patients is characterized by higher frequencies of pro-inflammatory B cell subsets as compared to that of E_H_ individuals

**FIGURE 3:**
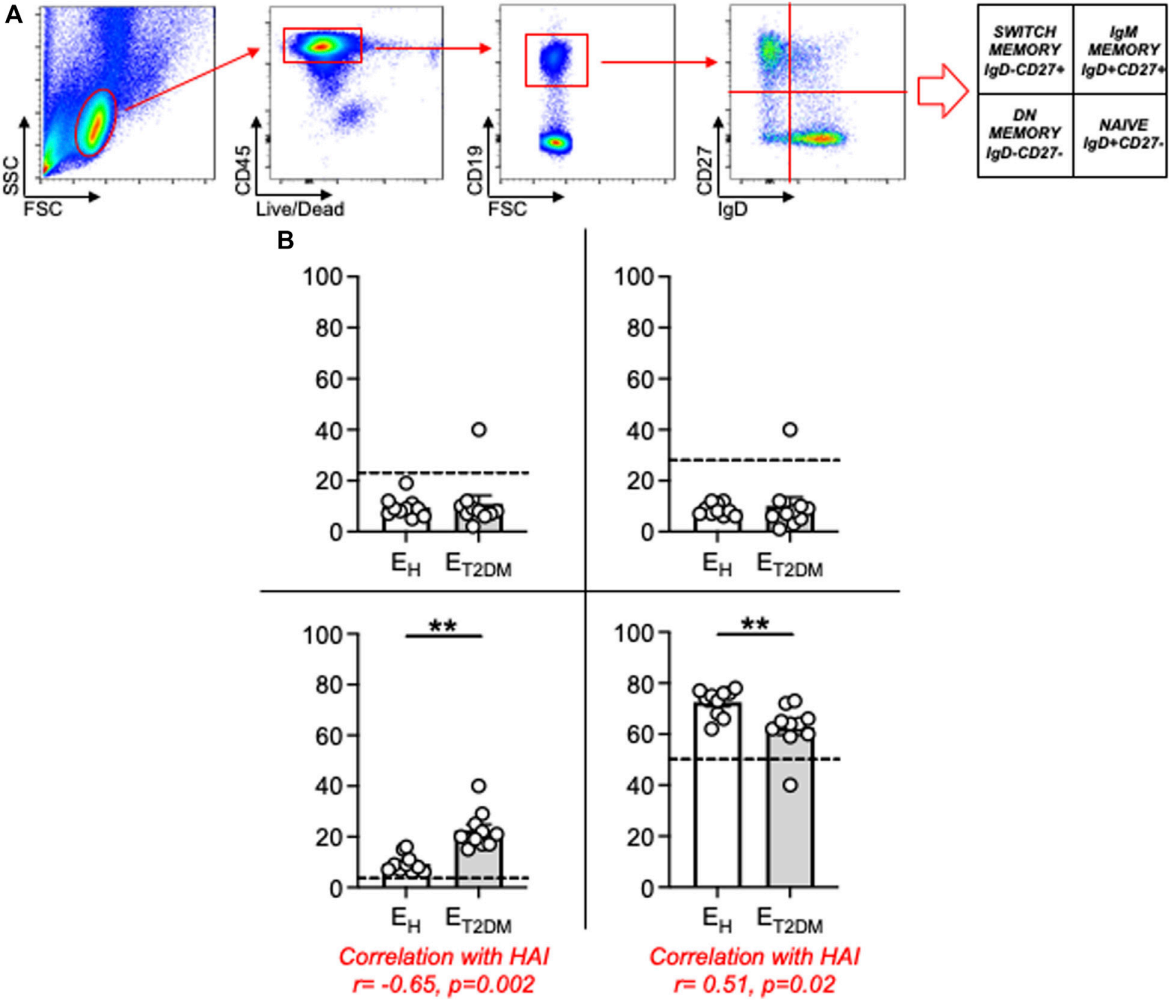
Increased blood frequencies of inflammatory DN B cells in the B cell pool of E_T2DM_ patients as compared to that of E_H_ individuals. **(A)** PBMCs were membrane stained with Live/Dead detection kit and anti-CD45/CD19/CD27/IgD antibodies. Gating strategies to evaluate the major B cell subsets are shown. **(B)** Frequencies of naïve (lower right quadrant), IgM memory (upper right quadrant), switched memory (upper left quadrant) and DN (lower left quadrant) B cells from all donors (means ± SE), gated on live CD45^+^ CD19^+^. In each graph, dotted lines indicate values in young individuals (naïve: 48.2 ± 1.4; IgM memory: 25.8 ± 3.1; switched memory: 23.3 ± 2.9; DN: 2.8 ± 0.5). Mean comparisons between groups were performed by unpaired Student’s t test (two-tails). **p < 0.01.

Work from different groups including ours has established that the age-related redistribution of B cell subsets in the circulating B cell pool influences the serum antibody response to the influenza vaccine (Frasca et al., 2016b; Wagner et al., 2018; Frasca et al., 2019; Frasca and Blomberg, 2020; Frasca et al., 2021a). This redistribution is characterized by the expansion of the most pro-inflammatory B cell subset, called Double Negative (DN) B cells, which also expands in the blood of individuals with inflammatory conditions and diseases such as obesity (Alberti et al., 1998; Bonomini et al., 2015; Berbudi et al., 2020), autoimmune diseases (van der Geest et al., 2016; Frasca et al., 2021b; Verschoor et al., 2022), and both chronic (Frasca et al., 2016a; Ferlita et al., 2019; Frasca et al., 2021c) and acute (Xiang et al., 2023) infectious diseases. Our results herein in Figure 3 confirm these previously published results. Figure 3A shows gating strategies to evaluate the different B cell subsets after staining PBMC with a Live/Dead kit, anti-CD45, anti-CD19, anti-CD27 and anti-IgD antibodies to evaluate the frequencies of the major B cell subsets. Results from all the participants are shown in Figure 3B. Briefly, only two subsets show significant age-associated changes, naive B cells which are significantly decreased, and DN B cells which are significantly increased. As expected, naïve B cell frequencies are even more decreased, and DN B cells even more increased, in E_T2DM_ patients versus E_H_ controls. No changes on IgM memory or switch memory B cells were observed. We have previously demonstrated that the expansion of DN B cells with age is linked to decreased protective antibodies to the influenza vaccine (Frasca et al., 2016b; Frasca et al., 2019; Frasca and Blomberg, 2020; Frasca et al., 2021a). Results were confirmed also in this cohort, and correlations are shown in Figure 3B.

Results in Figures 1–3 altogether demonstrate that the inflammatory status of an individual is strictly linked to the intrinsic inflammatory status of his/her B cells, both influencing the capacity to respond to the influenza vaccine.

### Increased secretion of the pro-inflammatory cytokines IL-6 and TNF-α, and decreased secretion of the anti-inflammatory cytokine IL-10, after *in vitro* stimulation of B cells from E_T2DM_ patients as compared to those from E_H_ individuals

**FIGURE 4:**
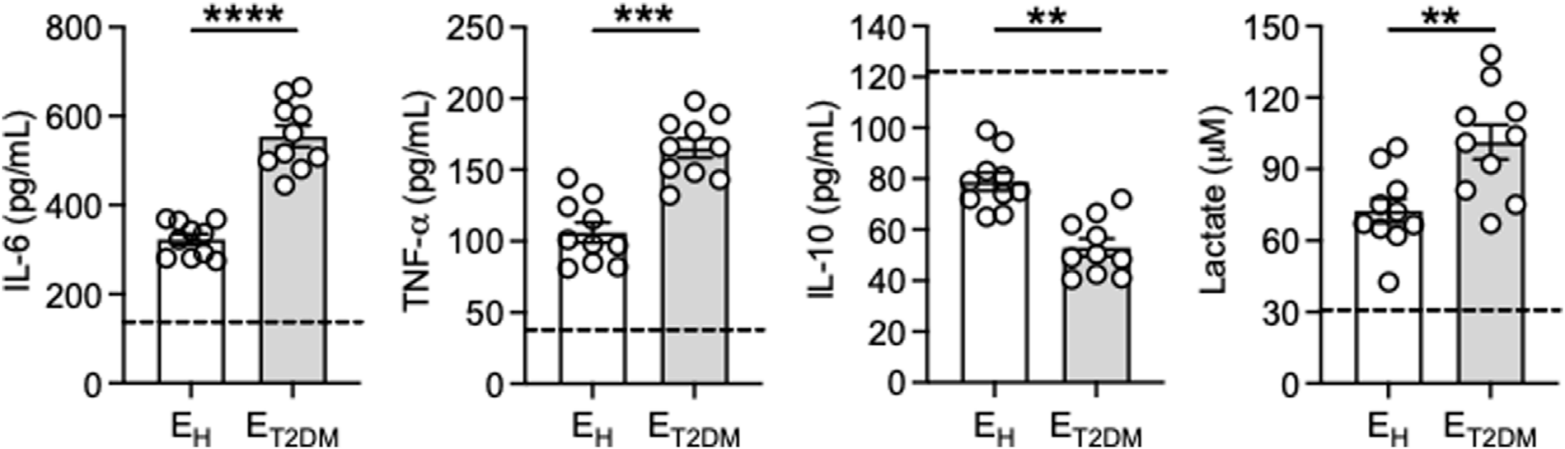
Increased secretion of the pro-inflammatory cytokines IL-6 and TNF-α, and decreased secretion of the anti-inflammatory cytokine IL-10, after *in vitro* stimulation of B cells from E_T2DM_ patients as compared to those from E_H_ individuals. B cells were isolated from PBMCs using CD19 microbeads and positive selection. B cells (10^6^ cells/mL) were cultured with CpG for 2 days. IL-6, TNF-α and IL-10 were measured in culture supernatants by CBA, lactate was measured by ELISA. Dotted lines indicate values in young individuals which were 150 ± 26 (IL-6), 41 ± 3 (TNF-α), 121 ± 19 (IL-10), 31 ± 2 (lactate). Mean comparisons between groups were performed by unpaired Student’s t test (two-tails). ***p < 0.001, ****p < 0.0001.

To further confirm the higher inflammatory profile of the B cell pool of E_T2DM_ patients versus that of E_H_ controls, we measured *in vitro* secretion of the pro-inflammatory cytokines TNF-α and IL-6 in culture supernatants of CpG-stimulated B cells from the three groups of individuals. Briefly, B cells were isolated from the blood using magnetic sorting and then stimulated for 2 days with CpG, then supernatans were collected and evaluated for the presence of IL-6, TNF-α and IL-10 by flow cytometry and CBA, as well as the secretion of the metabolite lactate by ELISA. Results in Figure 4 show higher secretion of IL-6, TNF-α and lactate in B cell supernatants from elderly individuals as compared to those from young controls (dotted line), and secretion was higher in B cells from E_T2DM_ patients as compared to those from E_H_ individuals. IL-10, conversely, was found decreased by age, as previously reported (van der Geest et al., 2016), and also decreased in B cells from E_T2DM_ patients versus those from E_H_ individuals.

Because we have shown above that the B cell pool of E_T2DM_ patients has more pro-inflammatory B cell subsets as compared to that of E_H_ individuals, we wanted to investigate the contribution of the DN B cell subset, that expands in E_T2DM_ patients, to the secretion of pro- and anti-inflammatory cytokines. We sorted DN B cells, we stimulated them in culture for 48 h with CpG, we collected the supernatant and we measured cytokine secretion by CBA. Results in Table 1 show higher amounts of IL-6, TNF-α and lactate, whereas IL-10 was undetectable, in B cells from E_T2DM_ patients as compared to those from E_H_ controls. These results confirm that the subset of DN B cells is the most inflammatory in the B cell pool.

**TABLE 1:** Secretion of pro- and anti-inflammatory cytokines and metabolites in culture supernatants of sorted DN B cells from E_H_ individuals and E_T2DM_ patients.

Cytokine/metabolite	E_H_	E_T2DM_
IL-6 (pg/mL)	240 ± 15	383 ± 40*
TNF-α (pg/mL)	80 ± 1	135 ± 8***
Lactate (μM)	58 ± 3	83 ± 7*

Results are from four individuals/group.

IL-6, TNF-α and IL-10 were measured by CBA, lactate was measured by ELISA.

*p < 0.05, ***p < 0.001.

Correlation between IL-6 and HAI: r = −0.79, *p* = 0.02.

Correlation between TNF-α and HAI: r = −0.82, *p* = 0.01.

Correlation between lactate and HAI: r = −0.77, *p* = 0.03.

These results confirm previous observations demonstrating that B cells contribute to the establishment and maintenance of inflammaging through the secretion of pro-inflammatory cytokines. This function is exacerbated in B cells from elderly individuals and especially in those with age-associated conditions and diseases such as T2DM. B cell-derived inflammatory cytokines, as we have already demonstrated in mice (Li et al., 2023) and humans (medRxiv [Preprint]. 2023 August 9:2023.08.07.23293760. doi: 10.1101/2023.08.07.23293760), also influence the differentiation of different T cell subsets, such as TH1 and TH17, which secrete additional pro-inflammatory mediators, also contributing to inflammaging. The contribution of B cell-derived pro-inflammatory cytokines becomes even more critical in inflamed tissues (obese adipose tissue, tumor microenvironment, synovial fluid of rheumatoid arthritis patients, atherosclerotic plaques), as well as in the blood of patients with autoimmune diseases, where their concentrantion rises significantly, increasing local inflammatory reactions, adverse effects and pathogenicity.

### B cells from E_T2DM_ patients show higher mitochondrial function than those from E_H_ individuals

**FIGURE 5:**
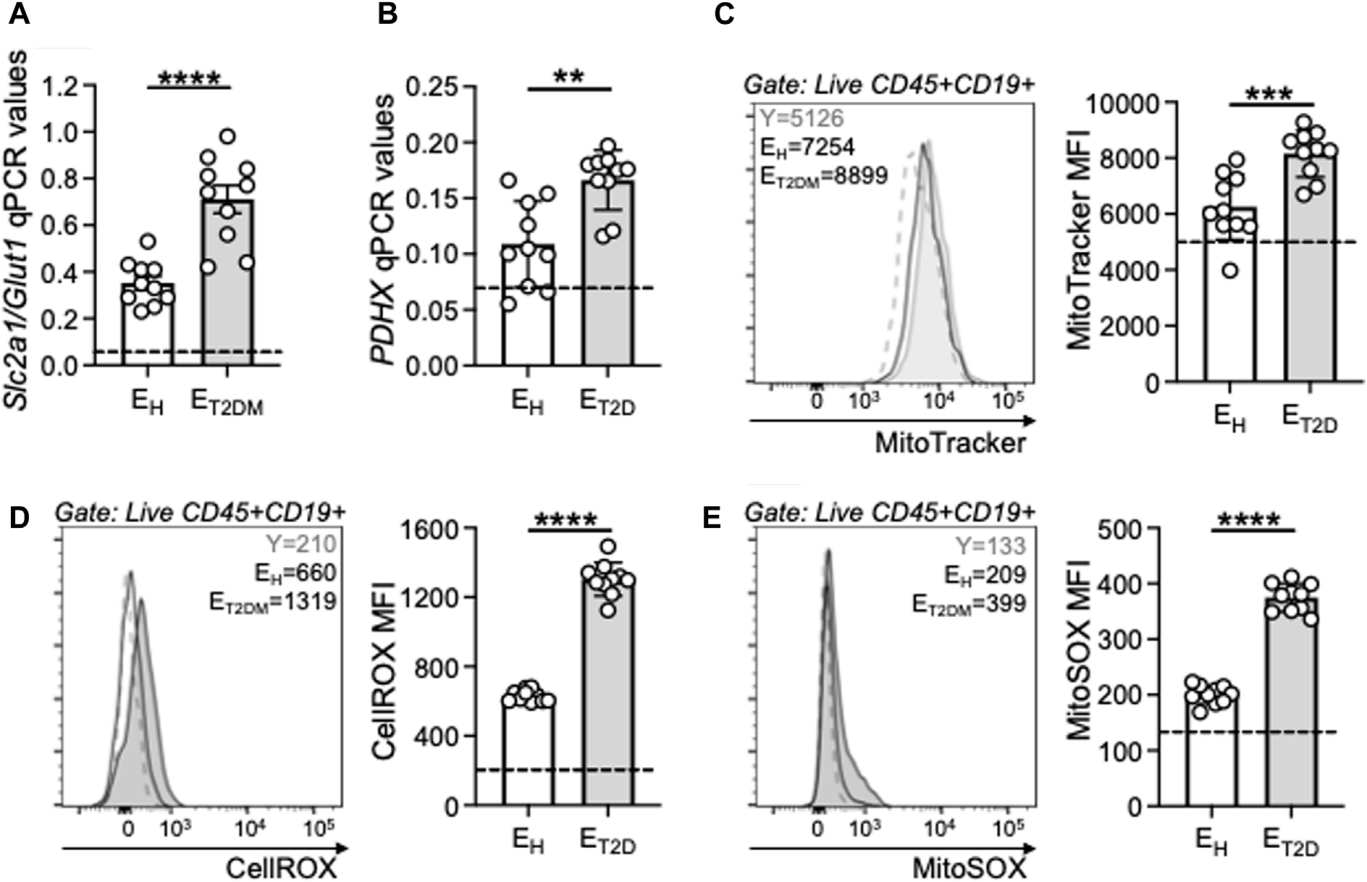
B cells from E_T2DM_ patients show higher mitochondrial function than those from E_H_ individuals. B cells were isolated from PBMCs using CD19 microbeads and positive selection, and left unstimulated. The mRNA was extracted and qPCR reactions ran to evaluate expression of transcripts for Glut 1 (SLC2A1) **(A)** and PDHX **(B)**. Results show qPCR values (2^−ΔCt^). **(C)** Mitochondrial mass staining using the MitoTracker Green probe. Representative flow cytometry histograms from one young (dotted line), one E_H_ and one E_T2DM_ individual (left) and MitoTracker Green MFI results from all individuals (right). **(D)** Cytosolic ROS measured by flow cytometry using the fluorescent CellROX reagent. Representative flow cytometry histograms from one young (dotted line), one E_H_ and one E_T2DM_ individual (left) and cytoplasmic ROS MFI results from all individuals (right). **(E)** Mitochondrial ROS measured by flow cytometry using the fluorescent MitoSOX reagent. Representative flow cytometry histograms from one young (dotted line), one E_H_ and one E_T2DM_ individual (left) and MitoSOX MFI results from all individuals (right). Dotted lines indicate values in young individuals (Glut 1: 0.07 ± 0.01; PDHX: 0.07 ± 0.02; MitoTracker MFI: 5,100 ± 48; CellROX MFI: 210 ± 18; MitoSOX; 135 ± 11). Mean comparisons between groups were performed by unpaired Student’s t test (two-tails). **p < 0.01, ***p < 0.001, ****p < 0.0001.

Our previously published results in mice (Frasca et al., 2021b) and humans (Frasca et al., 2019; Frasca et al., 2021a; Frasca et al., 2021c) have shown that the intrinsic inflammatory profile of B cells is associated with a higher metabolic status, which is needed to support the secretion of inflammatory mediators, and is characterized by higher RNA expression of key metabolic enzymes involved in glucose uptake and glycolytic pathways. Therefore, we measured the metabolic profile of B cells from the same three groups of participants by evaluating mRNA expression of the glucose transporter Glut1 (SLC2A1), the major glucose transporter of blood B cells, and of Pyruvate DeHydrogenase X (PDHX), a member of the PDH complex that converts pyruvate into ATP and represents a measure of oxidative phosphorylation (OXPHOS), mitochondrial function and Reactive Oxygen Species (ROS) production. Results in Figure 5 show increased expression of transcripts for the glucose transporter Glut1 (SLC2A1, A) and PDHX (B) in B cells from E_T2DM_ patients as compared to those from E_H_ individuals. B cells from E_T2DM_ patients are also characterized by higher mitochondrial mass, as evaluated by flow cytometry using the mitochondrial probe MitoTracker Green (C), cytosolic ROS, as evaluated by flow cytometry and CellROX staining (D) and mitochondrial ROS measured by flow cytometry and MitoSOX staining (E). Cytosolic ROS and mitochondrial ROS show similar differences in B cells from E_T2DM_ patients as compared to those from E_H_ individuals, and the two measures are significantly correlated (Pearson’s r = 0.96, *p* < 0.0001).

Mitochondrial dysfunction and excess of ROS production are detrimental to immune cell function and they have been shown to be associated with the age-related impairment of immune responses, susceptibility to infections and reduced vaccine responses (McGuire, 2019; Maldonado et al., 2023). Our results herein confirm these findings, and we show a negative association between the influenza vaccine response and the mitochondrial markers evaluated above (Table 2). Our results demonstrate that the decline in humoral immunity to the influenza vaccine is at least in part related to the intrinsic inflammatory status of B cells, which is metabolically supported.

**TABLE 2:** Correlations between vaccine responses and mitochondrial measures in B cells from E_H_ individuals and E_T2DM_ patients.

Correlation	Person’s r	P-value
HAI and Glut1	−0.69	**0.0008**
HAI and PDHX	−0.29	0.07
HAI and MitoTracker	−0.52	**0.02**
HAI and CellROX	−0.82	**<0.0001**
HAI and MitoSOX	−0.78	**<0.0001**

Significant correlations are in bold.

Mitochondrial dysfunction is also known to be associated with insulin resistance (Lowell and Shulman, 2005) and therefore implicated not only in T2DM development, but also in its progression and deterioration (Pinti et al., 2019). It is also known that dynamic changes in mitochondrial morphology are associated with hyperglycemia-driven ROS production (Yu et al., 2006), which is known as a major cause of the clinical complications associated with T2DM (Brownlee, 2001). Although the interplay of mitochondrial dysfunction with oxidative stress and inflammation in T2DM is well known, little is known about the contribution of immune cells in these processes. Here we show that B cells are highly inflammatory and highly metabolic in E_H_ individuals, and even more in E_T2DM_ patients, providing examples of how metabolic pathways can represent potential novel therapeutic targets in healthy aging and in aging with associated diseases such as T2DM. Our results on B cell metabolic imbalance and mitochondrial dysregulation strongly support the contribution of B cells to the overall inflammatory status of T2DM patients, ROS production and associated oxidative capacities leading to tissue damage and disease complications.

## Conclusion

Results herein show that E_T2DM_ patients are highly inflamed as compared to E_H_ controls, and are characterized by decreased antibody responses to the influenza vaccine, primarily because their B cell pool is enriched in pro-inflammatory B cell subsets that secrete inflammatory products that contribute to systemic inflammation. To perform this function, B cells are metabolically supported. The findings presented in this paper altogether highlight the relationship between B cell metabolic status and intrinsic inflammation in E_H_ as well as in E_T2DM_ patients.

The increased prevalence of T2DM in the elderly, either lean or with overweight/obesity, indicates the need of deep understanding of the molecular changes which may represent therapeutic targets to prevent the pathological effects of mitochondrial dysfunction not only in T2DM but also in other diseases associated with hyperglycemia.

## Data Availability

The original contributions presented in the study are included in the article/supplementary material, further inquiries can be directed to the corresponding authors.
